# Antiretroviral Resistance and Pregnancy Characteristics of Women with Perinatal and Nonperinatal HIV Infection

**DOI:** 10.1155/2016/4897501

**Published:** 2016-06-19

**Authors:** Gweneth B. Lazenby, Okeoma Mmeje, Barbra M. Fisher, Adriana Weinberg, Erika K. Aaron, Maria Keating, Amneris E. Luque, Denise Willers, Deborah Cohan, Deborah Money

**Affiliations:** ^1^Department of Obstetrics and Gynecology, Medical University of South Carolina, 96 Jonathan Lucas Street, Suite 624, Charleston, SC 29425, USA; ^2^Department of Obstetrics and Gynecology, University of Michigan, L4100 Women's Hospital, 1500 East Medical Center Drive, Ann Arbor, MI 48109, USA; ^3^Department of Pediatrics, University of Colorado Anschutz Medical Campus, 12700 E. 19th Box B168, Aurora, CO 80045, USA; ^4^Department of Medicine, Drexel University, 1427 Vine Street, 2nd Floor, Philadelphia, PA 19102, USA; ^5^Department of Medicine, University of Rochester, 601 Elmwood Avenue, Box 689, Rochester, NY 14642, USA; ^6^Department of Obstetrics and Gynecology, Washington University, 4921 Parkview Place, St. Louis, MO 63110, USA; ^7^Department of Obstetrics and Gynecology, University of California, 350 Parnassus Avenue No. 908, San Francisco, CA 94117, USA; ^8^Department of Obstetrics and Gynecology, University of British Columbia, 1190 Hornby Street, 4th Floor, Vancouver, BC, Canada V6Z 2K5

## Abstract

*Objective*. To compare HIV drug resistance in pregnant women with perinatal HIV (PHIV) and those with nonperinatal HIV (NPHIV) infection.* Methods*. We conducted a multisite cohort study of PHIV and NPHIV women from 2000 to 2014. Sample size was calculated to identify a fourfold increase in antiretroviral (ARV) drug resistance in PHIV women. Continuous variables were compared using Student's *t*-test and Wilcoxon rank-sum tests. Categorical variables were compared using *χ* ^2^ and Fisher's exact tests. Univariate analysis was used to determine factors associated with antiretroviral drug resistance.* Results*. Forty-one PHIV and 41 NPHIV participants were included. Women with PHIV were more likely to have drug resistance than those with NPHIV ((55% versus 17%, *p* = 0.03), OR 6.0 (95% CI 1.0–34.8), *p* = 0.05), including multiclass resistance (15% versus 0, *p* = 0.03), and they were more likely to receive nonstandard ARVs during pregnancy (27% versus 5%, *p* = 0.01). PHIV and NPHIV women had similar rates of preterm birth (11% versus 28%, *p* = 0.08) and cesarean delivery (47% versus 46%, *p* = 0.9). Two infants born to a single NPHIV woman acquired HIV infection.* Conclusions*. PHIV women have a high frequency of HIV drug resistance mutations, leading to nonstandard ARVs use during pregnancy. Despite nonstandard ARV use during pregnancy, PHIV women did not experience increased rates of adverse pregnancy outcomes.

## 1. Introduction

Less than one percent of women living with HIV have perinatally acquired HIV infection (PHIV). According to recent Centers for Disease Control and Prevention (CDC) HIV surveillance data, approximately 2,388 PHIV women are living in the United States [1]. Due to lifelong HIV infection, many women with PHIV have been exposed to multiple antiretroviral (ARV) therapy regimens. These exposures may include inadequate therapy during periods when there were limited ARV therapy options, such as mono and dual therapy. As a consequence of suboptimal therapy, inconsistent drug adherence, and/or prolonged intermittent exposure to multiple ARV classes, PHIV women may have HIV that has developed significant drug resistance [2–5].

Antiretroviral drug resistance can limit options for therapy during pregnancy and potentially complicate obstetrical care for HIV-infected women. PHIV women who have developed ARV drug resistance may require ARV therapies which are less well studied in pregnancy and may have unknown toxicities [6, 7]. Potentially secondary to noncompliance or a suboptimal ARV regimen, PHIV patients may have poor viral suppression during pregnancy resulting in cesarean delivery and a higher risk of perinatal transmission [3, 4, 8, 9]. Examples of other risks specific to PHIV pregnant women include complex psychosocial issues, unplanned pregnancies, and transmission of HIV to susceptible partners [3, 6, 10, 11].

ARV resistance rates have been reported as high as 30–50% in PHIV pregnant women [4, 9, 12]. ARV resistance mutations and drug classes affected are not well described in previous studies of PHIV pregnant women [4, 9, 12]. The primary objective of this study was to determine if PHIV pregnant women are more likely than pregnant women with nonperinatal HIV infection (NPHIV) to have ARV drug resistance. We describe the ARV classes affected by HIV genotypic mutations in both PHIV and NPHIV women. Our secondary objective is to describe and compare potential adverse maternal and neonatal outcomes among PHIV and NPHIV pregnant women.

## 2. Materials and Methods

The primary objective of this study was to determine how much more likely PHIV women are to have HIV genotypic mutations that confer clinically significant ARV resistance during pregnancy compared to NPHIV women. Prior data indicated the probability of genotypic resistance to ARVs, specifically nonnucleoside reverse transcriptase inhibitors (NNRTIs), in drug naïve NPHIV pregnant women to be 13–17% [13]. Based on previous reports, PHIV women may have ARV resistance rates as high as 50%. We anticipated a noncollinear relationship between ARV resistance and timing of HIV infection. In order to demonstrate odds ratio of at least 4.0 (13%  ×  4 = 52%) for ARV resistance in PHIV pregnant women relative to NPHIV women, 41 cases (PHIV) and 41 controls (NPHIV) were required to reject the null hypothesis that this odds ratio equals one with a probability of 0.8. The Type I error probability associated with this test of the null hypothesis was 0.05. The study was not powered to determine differences in pregnancy outcomes between PHIV and NPHIV.

Because PHIV is a rare condition in pregnancy, a multisite, retrospective cohort study was conducted to enroll the necessary sample size of PHIV participants. To identify potential study sites, an email was sent to all providers participating in the Reproductive Infectious Diseases listserv (ReproIDHIV Listserv) [14]. Collaborators from twenty-two sites responded with interest and were provided a copy of the protocol. Seven sites at academic medical centers in British Columbia, California, Colorado, Missouri, New York, Pennsylvania, and South Carolina elected to participate and the study protocol was approved by the institutional review boards at each site (IRB #13184). The study was supported by the departmental resources of the investigators.

Pregnant women with PHIV who received prenatal care at any of the study sites from 2000 to 2014 were eligible for participation. A woman was considered to have PHIV if her HIV serostatus was confirmed and determined to be acquired from her biological, serostatus-confirmed HIV-infected mother in the absence of any other risk factors (i.e., blood transfusion). Investigators at all 7 sites were responsible for identifying PHIV women for the study. Control participants were identified as pregnant women with NPHIV receiving prenatal care during the study period at any study site. A woman was considered to have NPHIV if she was diagnosed with HIV at ≥ 11 years of age in the absence of questionable perinatal infection (HIV-infected mother) or other risk factors for childhood infection (breastfeeding from an HIV-infected mother or blood transfusion). NPHIV participants were selected based on a similar age to study participants (±1 year of age). Participants were age-matched in order to reduce an uneven distribution of age-related medical comorbidities, such as preexisting hypertension and diabetes, which may potentially impact pregnancy outcomes. The majority of NPHIV were identified by the Principal Investigator from one academic institution.

The medical records of participants were reviewed by site specific investigators to obtain data from maternal antepartum, intrapartum, and postpartum care. Variables of interest were collected for all participants when available. Maternal variables included age, race, ethnicity, marital status, insurer, current partner's HIV status, history of opportunistic infections, existing medical and psychiatric diagnoses, gestational age at entry into prenatal care and at delivery, HIV RNA viral load (copies/mL) and CD4 cell count (cells/mm^3^) at entry into prenatal care and at delivery, ARV regimens before, during, and after pregnancy, HIV genotypic mutations associated with clinical drug resistance, evidence of sexually transmitted infections ((STIs):* Neisseria gonorrhea*,* Chlamydia trachomatis*,* Trichomonas vaginalis*,* Treponema pallidum*, human papilloma virus, hepatitis B and C, and herpes simplex virus), number of prenatal visits, antepartum complications, intrapartum prophylaxis IV zidovudine (AZT) when indicated, mode of delivery, postpartum infections including chorioamnionitis, and birth outcomes (e.g., live birth, intrauterine fetal demise, and spontaneous or elective abortion). Maternal antepartum complications of interest were hypertensive disorders of pregnancy, diabetes, maternal infection(s), preterm labor, anemia, and fetal anomaly and/or aneuploidy. In participants who gave birth to a live infant, data were collected from neonatal records up to 18 months of age. Variables of interest included birth weight, Apgar scores, level of nursery admit, neonatal postexposure ARV prophylaxis, duration of ARV prophylaxis, and HIV status. Neonates were considered HIV-infected if at least two positive HIV DNA PCR tests were confirmed before 18 months of age [15]. To protect confidentiality, all participants' data were deidentified.

Study site investigators collected and managed data using REDCap*™* (Research Electronic Data Capture), a secure, web-based application designed to support data capture [16]. Statistical analysis was performed using SAS® 9.4 software (Cary, N.C.). Continuous variables were compared using Student's *t*-test (means) and Wilcoxon rank-sum tests (medians). Continuous variables were tested for normality, and medians were compared when data were not normally distributed. Categorical variables were compared using *χ* ^2^ and Fischer's exact tests. Univariate logistic regression was used to determine factors associated with the presence of ARV drug resistance.

## 3. Results

**Figure 1:**
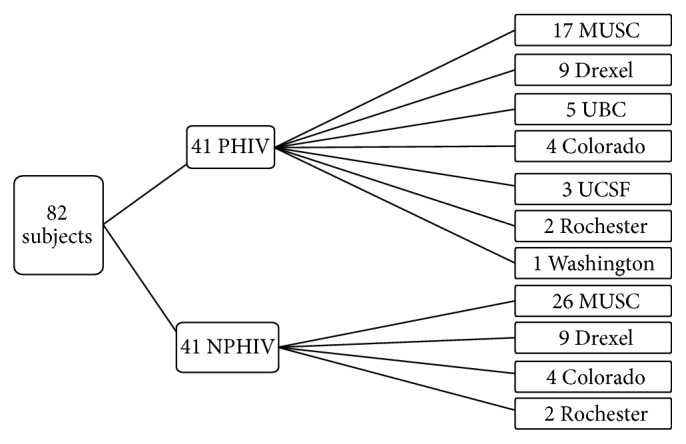
Site recruitment of pregnant women with perinatal and nonperinatal HIV infection. Site key: MUSC, Medical University of South Carolina, Charleston, SC; Drexel, Drexel University, Philadelphia, PA; UBC, University of British Columbia, Vancouver, BC; Colorado, University of Colorado, Aurora, CO; UCSF, University of California San Francisco, San Francisco, CA; Rochester, University of Rochester, Rochester, NY; Washington, Washington University, Saint Louis, MO.

As determined by the sample size calculation, 41 PHIV and 41 NPHIV women were included in the analysis (Figure 1). The mean age of participants at the time of pregnancy was 21 years (standard deviation (SD) ± 3) with a range of 14–30 years. The median parity of women was one (interquartile range (IQR), 0-1). The median gestational age at which women presented for prenatal care was 11 weeks (IQR, 7–14 weeks), and the mean number of prenatal visits prior to delivery was 10 (SD ± 5). The HIV status of the participants' male partner was recorded in approximately half of the women. NPHIV women were more likely to report an HIV-infected sexual partner(s) than PHIV women (32% versus 4%, *p* = 0.02). When comparing PHIV and NPHIV women, there were no differences in race, ethnicity, age, and prenatal care initiation or duration. NPHIV women were more likely to be parous than PHIV women (1 (IQR 0–4) versus 0 (IQR 0–2), *p* = 0.0004), and PHIV women were more likely to have a history of abnormal cervical cytology (50% versus 27%, *p* = 0.03). All pregnancies were singleton gestations (Tables 1 and 2).

The mean duration of known HIV infection for PHIV women was 21 (SD ± 4) years and it was 2 (SD ± 3) years for NPHIV women (*p* < 0.0001). Of the participants with NPHIV infection, 21 were diagnosed with HIV within one year of pregnancy and 20 were known to have HIV of duration of more than one year. Forty-three percent of all participants (36/82) reported current ARV use at initial presentation for prenatal care. PHIV women were more likely to report taking ARVs at presentation (68% versus 23%, *p* = 0.006). Although only 27 PHIV women were on ARVs at conception, all PHIV women in this study were exposed to ARVs during their lifetime prior to enrollment. The specific ARV regimens prescribed to individual subjects throughout their lives prior to enrollment in this study are not available for inclusion in this study. Of the NPHIV participants with HIV diagnosis greater than one year, only 30% reported current ARV use at initial presentation for prenatal care. The median HIV RNA viral load (copies/mL) and mean CD4 cell count (cells/mm^3^) collected within three months of the initial prenatal visit were not significantly different between PHIV and NPHIV women. Of the women reporting ARV use at their initial pregnancy visit, PHIV women were more likely than NPHIV women to have HIV RNA viral load ≥ 1,000 copies/mL (46% versus 0, *p* = 0.01) (Tables 1 and 2).

**Figure 2:**
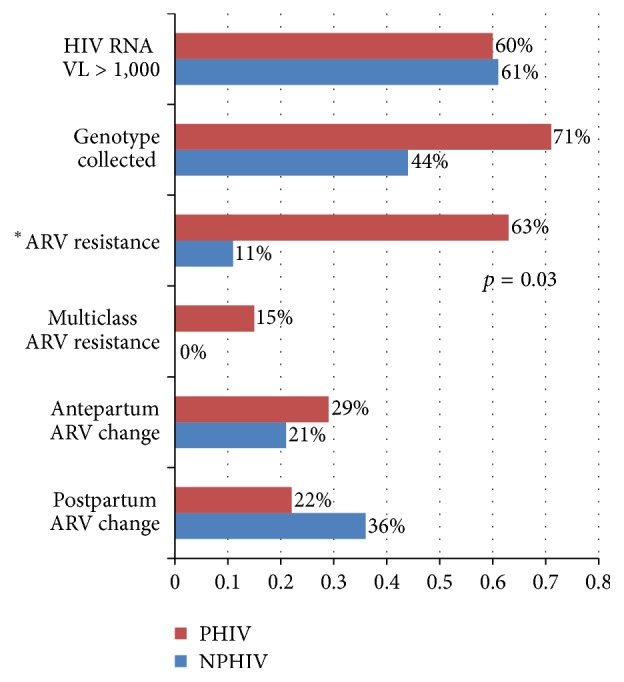
Antiretroviral drug resistance testing and therapy changes in women with perinatal and nonperinatal HIV infection. The proportion of women with a genotype collected and ARV resistance accounts for the number of participants eligible to have a genotype evaluated (HIV RNA viral load > 1,000 copies/mL). ^*∗*^ARV resistance and multidrug resistance rates were significantly different between groups.

Over half of participants (24 PHIV and 25 NPHIV) had an HIV RNA viral load ≥ 1,000 copies/mL at their initial prenatal evaluation. When using an HIV RNA viral load ≥ 1,000 copies/mL as criteria for collecting a genotype (HIV-1 genotype, ViroSeq®, ARUP laboratories, Salt Lake City, UT), 60% of participants would have been eligible for genotypic testing for HIV drug resistance. Not all participants who were eligible for resistance testing had a genotype collected within three months of their initial pregnancy visit. Collection of an HIV genotype during this time period was reported in 34 (42%) participants. Although similar numbers of PHIV and NPHIV women met criteria for resistance evaluation by genotype (24 PHIV and 25 NPHIV), PHIV participants were more likely to have genotypic testing collected within three months of their initial pregnancy visit compared to NPHIV counterparts (22 PHIV (54%) and 12 NPHIV (29%), *p* = 0.03). When accounting for genotype collection within three months of presentation for prenatal care, 55% PHIV versus 17% NPHIV had drug resistance (*p* = 0.03) (Figure 2).

In addition to genotype resistance noted in 12 PHIV and two NPHIV women within three months of initial prenatal care, ARV drug resistance was documented for seven additional participants either before pregnancy or during pregnancy. ARV drug resistance was documented in 21 participants (17 PHIV and four NPHIV), but 18 resistance patterns were available for analysis (15 PHIV and 3 NPHIV participants). Drug resistance to the NNRTI class was the most common mutation for both groups. Multiclass ARV drug resistance, resistance to more than one ARV class, occurred exclusively in PHIV women (16% versus 0, *p* = 0.03). Genotypic resistance to multiple ARV drug classes was documented in 6 PHIV women (NRTIs *n* = 6, NNRTIs *n* = 11, and protease inhibitors (PIs) *n* = 6). NPHIV women had resistance to NRTI (*n* = 1) and NNRTIs (*n* = 2), but no PIs resistance was noted. ARV regimens were adjusted during pregnancy in seven women (five PHIV and two NPHIV) secondary to drug resistance (Table 3).

Univariate analysis was performed to identify which maternal factors were associated with the ARV resistance mutations. Among women who had an HIV genotype collected, PHIV infection was associated with an increased risk of drug resistance (OR 6.0 (95% CI, 1.03–34.8), *p* = 0.05). PHIV infection was the only variable with a statistically significant association to ARV drug resistance. Other variables analyzed were the duration of HIV infection prior to pregnancy, elevated HIV viral load at presentation for pregnancy, medical comorbidities, race/ethnicity, history of psychiatric illness, and maternal age.

The majority of participants had documented ARV use during pregnancy (100% PHIV and 93% NPHIV, *p* = 0.24) (Table 2). PHIV women were more likely to take integrase inhibitors (20% versus 2%, *p* = 0.03). Fusion inhibitors (*n* = 3) and CCR5 antagonists (*n* = 1) were used exclusively in PHIV women. Resistance testing and results for these alternative drug classes were not available for analysis. At the time of delivery, there was no significant difference in the number of women from either group taking NRTIs, NNRTIs, and PIs (Table 3). An appendix in Supplementary Material available online at http://dx.doi.org/10.1155/2016/4897501 which provides a detailed description of the types of ARVs prescribed before, during, and after pregnancy for all study participants is available upon request.

Medical and psychiatric illnesses were common among study participants. Psychiatric illness was more common in women with PHIV (50% PHIV versus 27% NPHIV, *p* = 0.03). Depression was the most common psychiatric diagnosis, affecting 43% of PHIV and 22% of NPHIV women. NPHIV and PHIV women had similarly high rates of medical comorbidities in pregnancy (46% NPHIV versus 29% PHIV, *p* = 0.1). The most common medical comorbidities reported were asthma, obesity, chronic hypertension, and anemia. Only PHIV women had prior history of opportunistic infection(s). Rates of STIs were similar between PHIV and NPHIV women (*p* = 0.1). The following STIs were common among participants in both groups: genital herpes (68%),* T*.* vaginalis* (22%),* C*.* trachomatis* (14%), and* N*.* gonorrhoeae* (3%). Chronic hepatitis was infrequent among participants (6%); three women had hepatitis B and two had hepatitis C. Both groups had similar rates of pregnancy-related complications (45% versus 59%, *p* = 0.2). The most common complications of pregnancy were hypertensive disorders of pregnancy (12%), preterm labor/shortened cervical length/preterm delivery (20%), and premature rupture of membranes (2%) (Tables 1 and 2).

Live birth rates and cesarean delivery rates were similar among PHIV and NPHIV women ((97% PHIV versus 98% NPHIV, *p* = 1.0) and (47% PHIV versus 46% NPHIV, *p* = 0.9), resp.). At the time of delivery, the proportions of participants with HIV RNA viral load ≥ 1,000 copies/mL and HIV RNA viral loads below the level of detection (<40 copies/mL) were similar between groups ((26% PHIV versus 28% NPHIV, *p* = 0.9) and (56% PHIV versus 63% NPHIV, *p* = 0.5), resp.). The difference in the proportion of participants with HIV RNA viral loads between 40 and 999 copies/mL near delivery was not statistically significant (18% PHIV and 9% NPHIV, *p* = 0.18). Similar proportions of participants received at least three hours of intrapartum IV AZT when indicated (80% versus 84%, = 0.7) (Table 2).

The median gestational age at the time of delivery was 38 weeks in both groups. Preterm birth (<37 weeks gestation) rates were not significantly different between groups (11% PHIV versus 28% NPHIV, *p* = 0.08). Median birth weights were lower in the NPHIV women (2,742 (IQR 2,435–3,200) versus 3,065 (IQR 2,659–3,370), *p* = 0.02), but the proportion of low birth weight (<2,500 gm) infants was similar (34% NPHIV versus 22% PHIV, *p* = 0.2). Neonatal intensive care unit admissions were not significantly different between groups (29% NPHIV versus 14% PHIV, *p* = 0.2). The median duration of neonatal postexposure AZT prophylaxis was six weeks for both groups. HIV perinatal transmission was documented among two infants (2/32, 6%) born to a single NPHIV mother one year apart. The mother did not have prenatal care and did not take ARVs during either pregnancy. Both infants were born preterm, one by spontaneous vaginal delivery complicated by previable premature rupture of membranes and chorioamnionitis and the other by emergent cesarean delivery in the setting of abruption and preeclampsia. There were no documented cases of HIV perinatal transmission among women with PHIV (Table 2).

## 4. Conclusions

HIV genotypic patterns suggestive of clinically relevant ARV drug resistance were documented in PHIV pregnant women three times more frequently than NPHIV pregnant women. Previous studies have documented clinically relevant genotypic mutations in 30–50% of PHIV pregnant women and in 13–17% of NPHIV pregnant women [4, 9, 12]. We anticipated and found comparatively higher rates of ARV drug resistance among PHIV women likely due to their lifelong HIV infection and potential intermittent exposure to multiple ARV classes and suboptimal ARV regimens. Providers caring for HIV-infected pregnant women should be aware of the potential for high rates of ARV resistance among all pregnant women in this age group but especially among women born with HIV infection.

Compared to previous studies, we were able to document the patterns of HIV drug resistance in PHIV pregnant women. The genotypic resistance in NPHIV women was limited to the NRTI and NNRTI classes compared to multiple ARV classes in PHIV women. Six PHIV women had multidrug resistant HIV infection, and one PHIV women had resistance mutations to all three major ARV classes (NRTI, NNRTIs, and PIs). Due to multidrug resistance, PHIV women were more likely to be prescribed ARV combinations that are nonstandard regimens for the prevention of perinatal transmission [15]. During the study period, nonstandard ARV therapies prescribed for PHIV pregnant women included integrase inhibitors, fusion inhibitors, and CCR5 antagonists. Given the importance of HIV viral suppression during pregnancy, providers caring for pregnant women with PHIV should be familiar with the potential use and limitations of these alternative ARVs during pregnancy.

Our findings of high rates of ARV drug resistance support the recommendation for HIV genotype analysis in early pregnancy. Genotypic testing should be collected and assessed as early as possible in all HIV-infected women [15]. These tests should be repeated during later pregnancy in women with poorly suppressed HIV RNA viral loads. When resistance to standard ARV classes used for prevention of perinatal transmission is suspected, providers should consider alternative drug classes and order additional resistance testing for integrase inhibitors, fusion inhibitors, or CCR5 antagonists.

PHIV women were more likely to report ARV use at the time of pregnancy diagnosis, but overall use of ARVs prior to conception was low. Only 68% of PHIV women and even fewer NPHIV women (23%) were on ARV therapy at initial presentation for pregnancy care. Only 30% of NPHIV women diagnosed with HIV at least one year prior to pregnancy reported ARV use at presentation for care. In previous studies, PHIV women were more likely to have poor viral suppression during pregnancy, likely due to inconsistent drug adherence [3–5, 8]. In contrast to these reports, PHIV women in our study had similar rates of virological suppression and comparable CD4 cell counts to NPHIV women during pregnancy. Providers caring for reproductive age women with HIV infection should be aware of their pregnancy intentions in order to reduce the risk of perinatal HIV transmission from delayed exposure to effective ARV therapy before and during pregnancy. Women currently not seeking pregnancy should be offered effective contraception [17].

Although the reduction of perinatal transmission is a primary objective of prenatal care for HIV-infected women, pregnancy is also a time when HIV-infected women are examined frequently and can be screened for other significant medical comorbidities. Current or past psychiatric illness, especially depression, was more common among PHIV women (50%). Given the potential effects of depression on pregnancy, HIV-infected women should receive a multidisciplinary approach including psychiatric evaluation and support services [10, 18]. Additionally, PHIV women were more likely to have a history of abnormal pap smears. Frequent visits during pregnancy can provide adequate time for evaluation for cervical cancer risks among HIV-infected women.

Our study revealed that HIV-serodiscordance was very common among pregnant women and their partners. PHIV women were more likely to have HIV susceptible partners (96%) compared to NPHIV women (68%). This difference is likely explained by NPHIV women potentially having acquired HIV from their current partners, as compared to PHIV counterparts, who acquired HIV at birth. Prenatal providers should be aware of the potential risk of HIV transmission to HIV susceptible partners during pregnancy. In an effort to reduce the transmission of HIV to susceptible partners, providers should provide risk reduction counseling to all serodiscordant couples and frequent HIV testing of susceptible partners and evaluate partners for HIV preexposure prophylaxis [19].

The limitations of this study are related to the retrospective cohort design. This study design was necessary given the relative rarity of the exposure of PHIV in pregnancy. Data were not available for every variable for every participant. Genotypic testing was not available for all 82 participants. This can partially be explained by 33 (40%) women having HIV RNA viral loads < 1,000 copies/mL at the time of initial pregnancy care. It is unclear to the investigators why genotypes were not collected in all 49 women who were eligible for HIV drug resistance evaluation (HIV RNA viral load ≥ 1,000 copies/mL). Three genotype resistance patterns were not available for detailed review. Another limitation of our study is that we neglected planning for and carrying out collection of information regarding substance use, including tobacco, alcohol, and prescription and/or street drugs. A prospective study design and inclusion of all PHIV and NPHIV women presenting to any site during the study period would be the most effective means for evaluating the primary outcome and potential differences in pregnancy outcomes. However, given the rarity of PHIV during pregnancy, a prospective, multisite study would exceed the resources available.

The results of this study may not be applicable to all populations. The majority of study participants were identified at an academic center in the Southeast, where the Principal Investigator practices. According to the CDC, the Southeast has the 2nd highest prevalence of HIV infection in the United States. As of 2011, the Southeast had the highest number of new HIV infections and the largest proportion of individuals living with stage 3 HIV/AIDS [1]. Perinatal transmission rates are also high in this region [1]. Due to the increased rate of perinatal HIV transmissions, it is reasonable that the majority of cases and controls are clustered in this region due to prevalence of infection. However, the entirety of PHIV cases were distributed from multiple locations in North America suggesting this data has generalizability for many centers caring for PHIV women during pregnancy.

This is the largest single group of PHIV women studied in comparison to age-matched NPHIV controls. This study adds to the medical literature by describing the types of HIV ARV mutations that affect the care of HIV-infected women during pregnancy. Based upon the data presented here, obstetric providers of PHIV and NPHIV women should have a high suspicion for clinically relevant HIV drug resistance early in pregnancy in order to best select effective therapies for the prevention of perinatal HIV transmission. Lastly, PHIV women did not experience higher rates of perinatal complications and there were no perinatal HIV transmissions to PHIV-exposed infants. Although not powered to identify any significant differences in pregnancy outcomes, our findings suggest that PHIV women may have similar pregnancy outcomes compared to NPHIV. Further investigation is needed, but providers can offer some reassurance to women living with PHIV infection that they are likely to have pregnancy outcomes comparable to other HIV-infected pregnant women.

## Supplementary Material

The supplementary appendix provides detailed information on the antiretroviral regimens (ARVs) prescribed to each subject in the study. These include ARVs before, during, and after pregnancy.

## Figures and Tables

**Table 1:** Maternal characteristics of pregnant women with perinatal and nonperinatal HIV infection.

	Perinatal HIV (*n* = 41)	Nonperinatal HIV (*n* = 41)	*p* value
Mean age, (years)	20.9 (±3.2)	21.7 (±3.1)	0.2
Black/African American	21 (51%)	29 (71%)	0.07
Hispanic/Latino	5 (12%)	4 (10%)	1.0
Mean years living with HIV infection	20.5 (±3.5)	2.4 (±2.8)	<0.0001
Current HIV-infected sexual partner	1/24 (4%)	7/22 (32%)	0.02
Parity	0 (0-1)	1 (0–2)	0.0004
History of sexually transmitted infection(s) (STI)	22 (51%)	25 (61%)	0.5
History of abnormal pap smear	17/37 (46%)	8/38 (21%)	0.02
Hepatitis B and/or C coinfection	2 (5%)	3 (8%)	0.7
History of opportunistic infection(s)	7/40 (18%)	0	
History of a psychiatric illness, including depression	20/40 (50%)	11 (27%)	0.03
Medical comorbidity^a^	12 (29%)	19 (46%)	0.11
STI diagnosis during pregnancy	5 (13%)	11 (27%)	0.11

All denominators are *n* = 41 unless otherwise stated. Continuous variables are represented as means (±standard deviation) and medians (interquartile range). Continuous variables are compared using Student's *t*-test for means, pooled for equal variances and Wilcoxon rank-sum tests are used to compare medians.  Medical comorbidities excluding HIV, hepatitis, and psychiatric illness, including hypertensive disorders, asthma, anemia, cholelithiasis, transaminitis, obesity, and neuropathy^a^.

**Table 2:** Antepartum, intrapartum, and neonatal findings among pregnant women with perinatal HIV and nonperinatal HIV infection.

	Perinatal HIV (*n* = 41)	Nonperinatal HIV (*n* = 41)	*p* value
*Antepartum*			
Gestational age in weeks at initial obstetric visit^a^	11 (6–14)	11 (8–18)	0.2
ARV use at time of conception	27 (68%)	9 (23%)	<0.0001
Initial HIV RNA viral load (copies/mL) in pregnancy	19,945 (99–20,915)	4,800 (41–19,047)	0.8
Initial CD4 cell count (cells/mm^3^) in pregnancy	426 (±271)	516 (±212)	0.1
Number of obstetric visits prior to delivery	10.4 (±5.2)	9.9 (±5.3)	0.7
Pregnancy complications^b^	17/38 (45%)	24 (59%)	0.2
ARV use during pregnancy prior to delivery	41 (100%)	38 (93%)	0.2

*Intrapartum and postpartum*			
Gestational age at delivery	38 (38-39)	38 (34–39)	0.4
Preterm delivery (<37 weeks)	4/37 (11%)	11/39 (28%)	0.08
HIV RNA viral load (copies/mL) at delivery	40 (0–2,500)	0 (0–1,680)	0.3
HIV RNA viral load > 1,000 copies/mL at delivery	10 (26%)	11 (28%)	0.9
HIV RNA viral load < 40 copies/mL at delivery	23 (56%)	26 (63%)	0.5
CD4 cell count (cells/mm^3^) at delivery^a^	484 (265–612)	514 (373–646)	0.2
CD4 cell count (cells/mm^3^) below 200 at delivery	11 (27%)	4 (10%)	0.08
IV AZT administered at least 3 hours prior to delivery when indicated	28/35 (80%)	31/37 (84%)	0.7
Cesarean delivery	18/38 (47%)	18/39 (46%)	0.9
Any maternal intrapartum or postpartum infection	5/37 (14%)	7/38 (18%)	0.6

*Neonatal*			
Live birth	37/38 (97%)	39/40 (98%)	1.0
Birth weight (gm)^a^	3,065 (2,659–3,370)	2,742 (2,435–3,200)	0.02
Low birth weight (<2,500 gm)	8/37 (22%)	13/38 (34%)	0.2
NICU admission	5/35 (14%)	10/35 (29%)	0.2
Duration of postexposure prophylaxis with oral AZT (weeks)	6 (6)	6 (6)	0.6
Perinatal HIV infection	0/30	2/32 (6%)	0.5

All denominators are *n* = 41 unless otherwise stated. Continuous variables are represented as means (±standard deviation) and medians (interquartile range). Continuous variables are compared using Wilcoxon rank-sum tests to compare medians (Monte Carlo estimates were used to compare some medians^a^) and pooled Student's *t*-test is used for means. Pregnancy complications included hyperemesis gravidarum, urinary tract infection/pyelonephritis, hypertensive disorders of pregnancy, cervical incompetence, threatened preterm labor, abruption, preterm birth (<37 weeks), preterm rupture of membranes, and anemia^b^.

**Table 3:** Antiretroviral drug resistance patterns in HIV-infected pregnant women.

Subject number	NRTI resistance	NNRTI resistance	PI resistance	ARV regimen change during pregnancy	ART prior to delivery
PHIV *n* = 15	*N* = 6	*N* = 11	*N* = 6	*N* = 5	

1		✓		✓	AZT/3TC, DDI, NVP, NFV
3			✓		FTC/TDF, DRV, RIT
5	✓			✓	FTC/TDF, LPV/RIT
10			✓		ABC/AZT/3TC
12		✓	✓	✓	FTC/TDF, DRV, RIT, T-20
16			✓		FTC/TDF, LPV/RIT
28	✓	✓		✓	ABC/3TC, EFV, DRV, RIT
29		✓			ABC, TDF, LPV/RIT
46		✓			FTC/TDF, RAL
50		✓	✓		ATZ/3TC, LOP/RIT
58	✓	✓			ETV, DRV, RIT, RAL
60	✓	✓	✓	✓	TPV, RIT, MVC, T-20, DTG
77	✓	✓			FTC/TDF, EVG, COB
78	✓	✓			DRV, RIT, RAL, ETV
79		✓			DRV, RIT, FTC/TDF, ABC/3TC

NPHIV *n* = 3	*N* = 1	*N* = 2	*N* = 0	*N* = 2	

18		✓			AZT/3TC, LPV/RIT
25		✓		✓	AZT/3TC, RAL
56	✓				AZT/3TC, NFV

The genotypic mutation types were not recorded for 3 subjects: 2 PHIV (#80 and 81) and 1 NPHIV (#76). The ART regimens at delivery for these subjects were #80, FTC/TDF/RPV; #81, FTC/ETC/EVG/COB; and #76, ABC/3TC, ETV. #76 required a change in ART during pregnancy. ART, antiretroviral therapy; NRTI, nucleoside reverse transcriptase inhibitor; NNRTI, nonnucleoside reverse transcriptase inhibitor; PI, protease inhibitor; 3TC, lamivudine; ABC, abacavir; ATV, atazanavir; COB, cobicistat; DRV, darunavir; DTG, dolutegravir; EFV, efavirenz; ETV, etravirine; EVG, elvitegravir; FTC, emtricitabine; LPV, lopinavir; MVC, maraviroc; NFV, nelfinavir; NVP, nevirapine; TDF, tenofovir; RAL, raltegravir; RIT, ritonavir, RPV, rilpivirine; T-20, enfuvirtide.
