# Supplementary material for: Antiretroviral Resistance and Pregnancy Characteristics of Women with Perinatal and Nonperinatal HIV Infection
Source: Infect Dis Obstet Gynecol. 2016 Jun 19;2016:4897501. doi: 10.1155/2016/4897501 (PMC4930810; doi:10.1155/2016/4897501)
Supplement: Supplementary file 1 — The supplementary appendix provides detailed information on the antiretroviral regimens (ARVs) prescribed to each subject in the study. These include ARVs before, during, and after pregnancy. [file 4897501.f1.pdf]

Appendix 1. Combined antiretroviral therapy before, during, and after pregnancy in women with PHIV and NPHIV

Subject number	HIV source	Delivery year	cART at conception	ARV class(es) resistance	cART during pregnancy	cART at delivery	cART postpartum
1	PHIV	2002	AZT/3TC, NFV	NNRTI	AZT/3TC, NFV	**ddI, NVP, AZT/3TC, NFV**	AZT/3TC, NFV
2	PHIV	2006	TDF, 3TC, NPV, T-20		TDF, 3TC, NPV, T-20	TDF, 3TC, NPV, T-20	TDF, 3TC, NPV, T-20
3	PHIV	2010		PI	FTC/TDF,DRV, RIT	FTC/TDF,DRV, RIT	FTC/TDF,DRV, RIT
4	PHIV	2011	FTC/TDF,DRV, RIT		FTC/TDF,DRV, RIT	FTC/TDF,DRV, RIT	FTC/TDF,DRV, RIT
5	PHIV	2009	EFV/FTC/TDF	NRTI	**AZT/3TC, LPV/RIT**	**FTC/TDF, LPV/RIT**	FTC/TDF, LPV/RIT
6	PHIV	2010			x		
7	PHIV	2011	FTC/TDF, ATV, RIT, RAL		FTC/TDF, ATV, RIT, RAL	FTC/TDF, ATV, RIT, RAL	FTC/TDF, ATV, RIT, RAL
8	PHIV	2009	ABC/3TC, ATZ, RIT		ABC/3TC, ATZ, RIT	ABC/3TC, ATZ, RIT	ABC/3TC, ATZ, RIT
9	PHIV	2008			FTC/TDF, LPV/RIT	FTC/TDF, LPV/RIT	**FTC/TDF, ATV, RIT**
10	PHIV	2006	ABC/3TC/AZT	PI	ABC/3TC/AZT	ABC/3TC/AZT	ABC/3TC/AZT
11	PHIV	2008			AZT/3TC, LPV/RIT	AZT/3TC, LPV/RIT	**AZT/3TC, ATV, RIT**
12	PHIV	2009	FTC/TDF, ATV, RIT	NNRTI, PI	FTC/TDF, ATV, RIT	**FTC/TDF, DRV, RIT, T-20**	**FTC/TDF, DRV, RIT, RAL**
13	PHIV	2010			AZT/3TC, LPV/RIT	AZT/3TC, LPV/RIT	**DISCONTINUED**
14	PHIV	2008			AZT/3TC, LPV/RIT	AZT/3TC, LPV/RIT	AZT/3TC, LPV/RIT
15	PHIV	2005			AZT/3TC, LPV/RIT	AZT/3TC, LPV/RIT	AZT/3TC, LPV/RIT
16	PHIV	2008	EFV/FTC/TDF	PI	**FTC/TDF, LPV/RIT**	FTC/TDF, LPV/RIT	FTC/TDF, LPV/RIT
17	NPHIV	2010	FTC/TDF, LPV/RIT		FTC/TDF, LPV/RIT	FTC/TDF, LPV/RIT	FTC/TDF, LPV/RIT
18	NPHIV	2010		NNRTI	AZT/3TC, NPV, NFV	**AZT/3TC, RAL**	AZT/3TC, RAL
19	NPHIV	2011			AZT/3TC, LPV/RIT	AZT/3TC, LPV/RIT	AZT/3TC, LPV/RIT
20	NPHIV	2010	AZT/3TC, NFV		AZT/3TC, NFV	AZT/3TC, NFV	AZT/3TC, NFV
21	NPHIV	2007			AZT/3TC, NFV	AZT/3TC, NFV	AZT/3TC, NFV
22	PHIV	2012			AZT/3TC, LPV/RIT	AZT/3TC, LPV/RIT	AZT/3TC, LPV/RIT
23	NPHIV	2012			FTC/TDF/RPV	FTC/TDF/RPV	FTC/TDF/RPV
24	NPHIV	2004			AZT/3TC, NVP	AZT/3TC, NVP	AZT/3TC, NVP
25	NPHIV	2011		NNRTI	AZT/3TC, LPV/RIT	AZT/3TC, LPV/RIT	AZT/3TC, LPV/RIT
26	NPHIV	2006			AZT/3TC, NPV, NFV	AZT/3TC, NPV, NFV	AZT/3TC, NPV, NFV
27	PHIV	2006	3TC, TDF, ATV, RIT		3TC, TDF, ATV, RIT	3TC, TDF, ATV, RIT	3TC, TDF, ATV, RIT
28	PHIV	2010	ABC/ETC, NVP, DRV, RIT	NRTI,NNRTI	ABC/ETC, NVP, DRV, RIT	**ABC/3TC, EFV, DRV, RIT**	ABC/3TC, EFV, DRV, RIT
29	PHIV	2012	ABC, TDF, LPV/RIT	NNRTI	ABC, TDF, LPV/RIT	ABC, TDF, LPV/RIT	ABC, TDF, LPV/RIT
30	NPHIV	2008			AZT/3TC, LPV/RIT	AZT/3TC, LPV/RIT	**DISCONTINUED**
31	NPHIV	2009			AZT/3TC, LPV/RIT	**AZT/3TC, TDF, LPV/RIT**	**DISCONTINUED**
32	NPHIV	2000			AZT/3TC	AZT/3TC	AZT/3TC
33	NPHIV	2001			x		
34	NPHIV	2012			AZT/3TC, LPV/RIT	AZT/3TC, LPV/RIT	AZT/3TC, LPV/RIT
35	NPHIV	2008			AZT/3TC, LPV/RIT	AZT/3TC, LPV/RIT	**DISCONTINUED**
36	NPHIV	2011			AZT/3TC, LPV/RIT	AZT/3TC, LPV/RIT	
37	NPHIV	2010			AZT/3TC, LPV/RIT	AZT/3TC, LPV/RIT	
38	NPHIV	2004			x		**DISCONTINUED**
39	NPHIV	2008			x		
40	NPHIV	2006			AZT/3TC	**AZT/3TC, NFV**	**DISCONTINUED**

41	NPHIV	2005			AZT/3TC, NFV	AZT/3TC, NFV	**DISCONTINUED**
42	NPHIV	2005			AZT/3TC, NVP	AZT/3TC, NVP	AZT/3TC, NVP
43	NPHIV	2006			AZT/3TC, NVF	AZT/3TC, NVF	discontinued
44	PHIV	2011	ABC/3TC, ETV, RAL		ABC/3TC, ETV, RAL	**TDF, ABC/3TC, DRV, RIT**	TDF, ABC/3TC, DRV, RIT
45	PHIV	2011	FTC/TDF, ATV, RIT		FTC/TDF, ATV, RIT	FTC/TDF, ATV, RIT	FTC/TDF, ATV, RIT
46	PHIV	2010		NNRTI	FTC/TDF, RAL	FTC/TDF, RAL	FTC/TDF, RAL
47	PHIV	2011	FTC/TDF, ATV, RIT		FTC/TDF, ATV, RIT	FTC/TDF, ATV, RIT	FTC/TDF, ATV, RIT
48	PHIV	2005	FTC/TDF, LPV/RIT		FTC/TDF, LPV/RIT	FTC/TDF, LPV/RIT	FTC/TDF, LPV/RIT
49	PHIV	2003	D4T, ABC, NFV		D4T, ABC, NFV	D4T, ABC, NFV	D4T, ABC, NFV
50	PHIV	2011		NNRTI, PI	AZT/3TC, LPV/RIT	AZT/3TC, LPV/RIT	AZT/3TC, LPV/RIT
51	NPHIV	2008	FTC/TDF, ATV, RIT		FTC/TDF, ATV, RIT	**AZT/3TC, NFV**	**AZT/3TC, LPV/RIT**
52	NPHIV	2006			AZT/3TC, LPV/RIT	AZT/3TC, LPV/RIT	discontinued
53	NPHIV	2006			AZT/3TC, NFV	AZT/3TC, NFV	AZT/3TC, NFV
54	NPHIV	2008			AZT/3TC, LPV/RIT	AZT/3TC, LPV/RIT	AZT/3TC, LPV/RIT
55	NPHIV	2010			AZT/3TC, LPV/RIT	AZT/3TC, LPV/RIT	**ABC/3TC, ATV, RIT**
56	NPHIV	2007	FTC/TDF, ATV, RIT	NRTI	**AZT/3TC, NFV**	AZT/3TC, NFV	**FTC/TDF, ATV, RIT**
57	PHIV	2010	EFV/FTC/TDF		**AZT/3TC, LPV/RIT**	AZT/3TC, LPV/RIT	AZT/3TC, LPV/RIT
58	PHIV	2013		NRTI, NNRTI	ETV, DRV, RIT, RAL	ETV, DRV, RIT, RAL	ETV, DRV, RIT, RAL
59	PHIV	2013	EFV/FTC/TDF		EFV/FTC/TDF	**RPV/TDF/FTC**	RPV/TDF/FTC
60	PHIV	2013	FTC/TDF, DRV, RIT, RAL, MVC	NRTI, NNRTI, PI	**FTC/ETC/EVG/COB, MVC, ETV, DRV, RIT**	**TPV, RIT, DTG, T-20, MVC**	TPV, RIT, DTG, T-20, MVC
61	NPHIV	2013	EFV/FTC/TDF, ATZ, RIT		**FTC/TDF, ATV, RIT**	FTC/TDF, ATV, RIT	FTC/TDF, ATV, RIT
62	NPHIV	2013	FTC/TDF, DRV, RIT		FTC/TDF, DRV, RIT	FTC/TDF, DRV, RIT	FTC/TDF, DRV, RIT
63	PHIV	2013	FTC/TDF, DRV, RIT		FTC/TDF, DRV, RIT	FTC/TDF, DRV, RIT	FTC/TDF, DRV, RIT
64	NPHIV	2014	FTC/ETC/EVG/COB		FTC/ETC/EVG/COB	FTC/ETC/EVG/COB	FTC/ETC/EVG/COB
65	PHIV	2013	FTC/TDF, DRV, RIT		FTC/TDF, DRV, RIT	FTC/TDF, DRV, RIT	FTC/TDF, DRV, RIT
66	PHIV	2013			ABC/3TC, TDF, RPV	ABC/3TC, TDF, RPV	ABC/3TC, TDF, RPV
67	NPHIV	2012			FTC/TDF, ATV, RIT	FTC/TDF, ATV, RIT	FTC/TDF, ATV, RIT
68	NPHIV	2012	EFV/FTC/TDF		**FTC/TDF, ATV, RIT**	FTC/TDF, ATV, RIT	**FTC/TDF, RPV**
69	NPHIV	2012			AZT/3TC, LPV/RIT	AZT/3TC, LPV/RIT	AZT/3TC, LPV/RIT
70	NPHIV	2012			AZT/3TC, LPV/RIT	**ABC/3TC, ATZ, RIT**	**DISCONTINUED**
71	NPHIV	2010			ABC/3TC, ATZ, RIT	ABC/3TC, ATZ, RIT	**DISCONTINUED**
72	NPHIV	2010	ABC/3TC, ATZ, RIT		ABC/3TC, ATZ, RIT	ABC/3TC, ATZ, RIT	ABC/3TC, ATZ, RIT
73	NPHIV	2010			FTC/TDF,DRV, RIT	FTC/TDF,DRV, RIT	**DISCONTINUED**
74	PHIV	2011	AZT/3TC, LPV/RIT		AZT/3TC, LPV/RIT	AZT/3TC, LPV/RIT	**DISCONTINUED**
75	NPHIV	2011	ABC/3TC, ATZ, RIT		ABC/3TC, ATZ, RIT	ABC/3TC, ATZ, RIT	ABC/3TC, ATZ, RIT
76	NPHIV	2010			AZT/3TC, ETV	**FTC/TDF, ETV**	**ABC/3TC, ETV**
77	PHIV	2014		NRTI, NNRTI	FTC/ETC/EVG/COB	FTC/ETC/EVG/COB	FTC/ETC/EVG/COB
78	PHIV	2014	FTC/TDF, ATV, RIT	NRTI, NNRTI	**ETV, DRV, RIT, RAL**	ETV, DRV, RIT, RAL	ETV, DRV, RIT, RAL
79	PHIV	2014	FTC/TDF,DRV, RIT	NNRTI	**FTC/TDF, ABC/3TC, DRV, RIT**	**FTC/TDF,DRV, RIT**	**TDF, ABC/3TC, DRV, RIT**
80	PHIV	2014			FTC/TDF/RPV	FTC/TDF/RPV	FTC/TDF/RPV
81	PHIV	2014	FTC/TDF/RPV		FTC/TDF/RPV	**FTC/ETC/EVG/COB**	FTC/ETC/EVG/COB
82	PHIV	2014	FTC/TDF, ATV, RIT		FTC/TDF, ATV, RIT	FTC/TDF, ATV, RIT	FTC/TDF, ATV, RIT

Changes in combined antiretroviral therapy (cART) are bolded. 3TC = lamivudine; ABC = abacavir; ATV= atazanavir; COB=cobicistat; d4T= stavudine; DDI= didanosine; DRV = darunavir; DTG = dolutegravir; EFV = efavirenz; ETV= etravirine; EVG= elvitegravir; FTC= emtricitabine; LPV= lopinavir; MVC= maraviroc; NFV= nelfinavir; NVP= nevirapine; TDF=tenofovir; TPV= tipranavir; RAL = raltegravir; RIT=ritonavir, RPV = rilpivirine; T-20= enfuvirtide
